# Supplementary material for: Agricultural impacts on streams near Nitrate Vulnerable Zones: A case study in the Ebro basin, Northern Spain
Source: PLoS One. 2019 Nov 8;14(11):e0218582. doi: 10.1371/journal.pone.0218582 (PMC6839874; doi:10.1371/journal.pone.0218582)
Supplement: S1 File — Location of the sampling sites (Table A); Environmental variables studied at each site, and categories and codes used in the present study (Table B); Functional (biological and ecological) traits and categories studied according to Tachet et al. (2006) (Table C); Mean values (for four sampling dates) and ranges (i.e. minimum and maximum) of the physicochemical variables not included in Table 1 (Table D); Values of hydromorphological variables determined in the May sampling campaign and not included in Table 1 (components of IHF -River Habitat Index-, QBR -Riparian Forest Quality Index- and percentage of macrophytes cover) (Table E); Values of topographic, geologic and land cover variables not included in Table 1 (Table F); Density (ind/m2) of macroinvertebrate taxa at each site (Table G); Relative abundance of each macroinvertebrate functional trait at study sites (Table H). (DOCX) [file pone.0218582.s001.docx]

**Supporting information 1**

**Table A.** Location of the sampling sites.

|  | Acronym | Stream | Sub-basin | Basin | Coordinate X | Coordinate Y |
| --- | --- | --- | --- | --- | --- | --- |
| Group 1 | Enc | Encemero | Oja-Tirón | Ebro | 30T 492993 | 4704514 |
|  | R1 | Reláchigo | Oja-Tirón | Ebro | 30T 492086 | 4693920 |
|  | R2 | Reláchigo | Oja-Tirón | Ebro | 30T 496254 | 4700124 |
|  | R3 | Reláchigo | Oja-Tirón | Ebro | 30T 498094 | 4705139 |
|  | Y1 | Yalde | Najerilla | Ebro | 30T 527750 | 4686271 |
|  | Y2 | Yalde | Najerilla | Ebro | 30T 526875 | 4692538 |
| Group 2 | Ea | Ea | Oja-Tirón | Ebro | 30T 503396 | 4715620 |
|  | Z1 | Zamaca | Zamaca | Ebro | 30T 513813 | 4710439 |
|  | Z2 | Zamaca | Zamaca | Ebro | 30T 517465 | 4711587 |
|  | T1 | Tuerto | Najerilla | Ebro | 30T 516909 | 4697302 |
|  | T2 | Tuerto | Najerilla | Ebro | 30T 521244 | 4699772 |

**Table B.** Environmental variables studied in each site, categories and codes used in the present study.

| **Category** | **Description** | **Code** | **Methodology** |
| --- | --- | --- | --- |
| Physico- | Concentration of NO_3_^-^ (ppm) | NO3 | Ion cromatography |
| chemical | Concentration of NO_2_^-^ (ppm) | NO2 |  |
|  | Concentration of PO_4_^3-^ (ppm) | PO4 |  |
|  | Concentration of F^-^ (ppm) | F |  |
|  | Concentration of Cl^-^ (ppm) | Cl |  |
|  | Concentration of SO_4_^2-^ (ppm) | SO4 |  |
|  | Concentration of NH_4_^+^ (ppm) | NH4 |  |
|  | Concentration of Na^+^ (ppm) | Na |  |
|  | Concentration of K^+^ (ppm) | K |  |
|  | Concentration of Ca^2+^ (ppm) | Ca |  |
|  | Concentration of Mg^2+^ (ppm) | Mg |  |
|  | Temperature (ºC) | Tª | *In situ* |
|  | Conductivity (µS/cm) | Cond |  |
|  | Concentration of Dissolved Oxygen (mg/l) | DO |  |
|  | pH | pH |  |
| Hydro- | Component of QBR referred to riparian vegetation cover | QBR-cover | (Munné *et al.*, 2003) |
| morphological | Comp. of QBR referred to vegetation cover structure | QBR-struc |  |
|  | Comp. of QBR referred to cover quality | QBR-quali |  |
|  | Comp. of QBR referred to river channel alteration | QBR-natur |  |
|  | Total score of the Riparian Forest Quality Index | QBR-total |  |
|  | Comp. of IHF referred to hard substrate embeddedness in fine sediment | IHF-embed | (Pardo *et al.,* 2002) |
|  | Comp. of IHF referred to riffle frequency | IHF-riffle |  |
|  | Comp. of IHF referred to substrate composition | IHF-subst |  |
|  | Comp. of IHF referred to velocity/depth regime | IHF-veloc |  |
|  | Comp. of IHF referred to shading of river bed | IHF-shade |  |
|  | Comp. of IHF referred to heterogeneity components | IHF-heter |  |
|  | Comp. of IHF referred to aquatic vegetation cover | IHF-veget |  |
|  | Total score of the Habitat Fluvial Index | IHF-total |  |
|  | Macrophytes cover (%) | Macrophytes | Visually estimated |
| Geologic | Upstream calcareous rocks (%) | CALC | GIS |
|  | Upstream conglomerate rocks (%) | CONG |  |
|  | Upstream sedimentary rocks (%) | SDIM |  |
|  | Upstream siliceous rocks (%) | SLIC |  |
| Land cover | Upstream agricultural land (%) | AGR | GIS |
|  | Upstream broadleaf forest (%) | BLF |  |
|  | Upstream coniferous forest (%) | CNF |  |
|  | Upstream pasture (%) | PAS |  |
|  | Upstream plantations (%) | PLT |  |
|  | Upstream moors, heathland, scrub and shrubs (%) | SSH |  |
| Topographic | Valley Width Index: ratio of valley width to channel width. | VWI | GIS |

**Table C.** Functional (biological and ecological) traits and categories studied according to Tachet *et al*. (2006)

| **Biological trait** | **Category** | **Biological trait** | **Category** | **Ecological trait** | **Category** |
| --- | --- | --- | --- | --- | --- |
| Reproductive cycles | less than 1 | Food | microorg. + fine sediment | Microhabitat | boulders/cobbles/pebbles |
|  | 1 |  | detritus < 1mm |  | gravel |
|  | more than 1 |  | dead plant >= 1mm |  | sand |
| Respiration | tegument |  | living microphytes |  | silt |
|  | gill |  | living macrophytes |  | macrophytes |
|  | plastron |  | dead animal >= 1mm |  | microphytes |
|  | spiracle |  | living microinvertebrates |  | twigs/roots |
|  | hydrostatic vesicle |  | living macroinvertebrates |  | organic detritus/litter |
| Locomotion and | flier |  | vertebrates |  | mud |
| substrate relation | surface swimmer | Feeding habits | absorber | Trophic status | oligotrophic |
|  | full water swimmer |  | deposit feeder |  | mesotrophic |
|  | crawler |  | shredder |  | eutrophic |
|  | burrower |  | scraper |  |  |
|  | interstitial |  | filter-feeder |  |  |
|  | temporarily attached |  | piercer |  |  |
|  | permanently attached |  | predator |  |  |
|  |  |  | parasite |  |  |

**Table D.** Average values (four sampling dates) and range of values of physicochemical variables not included in Table 1.

|  |  | Tª | Cond | NO2 (ppm) | F (ppm) | Cl (ppm) | SO4 (ppm) |
| --- | --- | --- | --- | --- | --- | --- | --- |
| Group 1 | Enc | 13.30 (6.80-17.20) | 2078 (1848-2380) | 0.030 (0.004-0.083) | 0.386 (0.268-0.491) | 44.90 (40.53-50.82) | 896.8 (789.4-1040.7) |
|  | R1 | 9.70 (5-90-13.60) | 208 (195-230) | 0.033 (0.027-0.037) | 0.055 (0.040-0.085) | 2.49 (2.28-2.91) | 5.4 (2.0-10.6) |
|  | R2 | 10.37 (8.10-12.20) | 423 (350-536) | 0.033 (0.027-0.035) | 0.042 (0.023-0.067) | 9.28 (8.05-10.48) | 40.6 (33.7-42.2) |
|  | R3 | 11.25 (6.10-16.30) | 1120 (770-1665) | 0.028 (0.018-0.025) | 0.071 (0.048-0.102) | 50.66 (30.57-79.76) | 334.5 (160.7-680.8) |
|  | Y1 | 12.05 (7.80-17.70) | 227 (208-246) | 0.035 (0.016-0.050) | 0.037 (0.026-0.052) | 32.52 (12.08-63.82) | 7.6 (3.2-14.3) |
|  | Y2 | 12.58 (10.10-15.20) | 466 (420-515) | 0.045 (0.028-0.082) | 0.034 (0.023-0.041) | 6.30 (4.91-9.09) | 16.9 (9.7-34.3) |
| Group 2 | Ea | 13.50 (5.90-20.20) | 1186 (865-1360) | 0.065 (0.016-0.091) | 0.039 (0.035-0.042) | 50.25 (25.99-82.16) | 305.4 (166.4-421.5) |
|  | Z1 | 13.23 (9.20-17.90) | 1061 (945-1204) | 0.045 (0.018-0.066) | 0.051 (0.035-0.070) | 65.04 (63.81-64.40) | 197.8 (187.0-223.8) |
|  | Z2 | 13.55 (9.60-19.30) | 1067 (937-1205) | 0.043 (0.011-0.090) | 0.057 (0.043-0.091) | 72.00 (63.56-87.16) | 204.4 (184.2-247.7) |
|  | T1 | 15.48 (8.30-21.90) | 883 (513-1300) | 0.578 (0.331-0.922) | 0.132 (0.045-0.343) | 34.08 (12.21-82.66) | 352.9 (131.5-807.6) |
|  | T2 | 12.95 (3.90-22.50) | 1363 (675-1950) | 0.453 (0.073-1.283) | 0.222 (0.087-0.435) | 48.87 (15.09-97.97) | 558.4 (236.7-989.9) |

|  |  | Na (ppm) | K (ppm) | Ca (ppm) | Mg (ppm) |
| --- | --- | --- | --- | --- | --- |
| Group 1 | Enc | 47.26 (38.90-56.12) | 7.67 (6.56-8.34) | 575.53 (540.15-596-31) | 95.08 (84.70-90.16) |
|  | R1 | 6.89 (6.47-7.62) | 0.51 (0.40-0.71) | 37.75 (35.20-39.21) | 5.28 (4.74-5.97) |
|  | R2 | 11.18 (9.65-12.79) | 2.19 (2.04-2.28) | 66.02 (55.51-71.53) | 10.79 (9.02-12.05) |
|  | R3 | 32.67 (20.47-51.20) | 3.46 (2.78-4.29) | 210.22 (116.74-374.75) | 56.48 (29.24-107.68) |
|  | Y1 | 26.72 (16.70-33.72) | 0.69 (0.30-1.72) | 40.20 (38.87-41.78) | 1.30 (1.00-1.61) |
|  | Y2 | 5.14 (4.53-6.74) | 1.22 (0.46-1.53) | 70.06 (65.49-75.04) | 5.95 (5.29-7.09) |
| Group 2 | Ea | 33.93 (24.28-48.51) | 4.90 (2.90-6.63) | 163.83 (117.06-207.12) | 57.24 (39.04-81.13) |
|  | Z1 | 27.67 (27.31-28.03) | 4.72 (3.92-6.89) | 152.57 (141.88-161.47) | 41.11 (40.23-42.15) |
|  | Z2 | 31.68 (30.28-34.21) | 4.86 (4.47-5.23) | 147.54 (142.47153.03) | 42.48 (39.66-47.68) |
|  | T1 | 12.67 (8.87-18.02) | 8.66 (5.58-11.85) | 160.85 (100.54-231.60) | 26.07 (16.30-38.47) |
|  | T2 | 32.61 (14.40-53.66) | 8.72 (4.26-13.21) | 229.17 (134.60-327.42) | 57.55 (26.91-92.09) |

**Table E.** Values of hydromorphological variables determined in May sampling and not included in Table 1 (components of IHF -River Habitat Index-, QBR -Riparian Forest Quality Index- and percentage of macrophytes cover). Possible highest punctuation for each component of IHF and QBR index are shown in parentheses. Description of each component are shown in S1 Table.

|  |  | IHF-embed  (10) | IHF-riffle  (10) | IHF-subst  (20) | IHF-veloc  (10) | IHF-shade  (10) | IHF-heter  (10) | IHF-veget  (30) | QBR-cover  (25) | QBR-struc  (25) | QBR-quali  (25) | QBR-natur  (25) | Macrophytes  (%) |
| --- | --- | --- | --- | --- | --- | --- | --- | --- | --- | --- | --- | --- | --- |
| Group 1 | Enc | 5 | 6 | 17 | 6 | 5 | 8 | 20 | 0 | 15 | 25 | 10 | 50 |
|  | R1 | 5 | 10 | 17 | 6 | 10 | 6 | 20 | 15 | 25 | 25 | 10 | 5 |
|  | R2 | 5 | 8 | 14 | 6 | 10 | 8 | 20 | 0 | 15 | 25 | 10 | 20 |
|  | R3 | 5 | 4 | 17 | 6 | 5 | 8 | 15 | 0 | 15 | 15 | 10 | 50 |
|  | Y1 | 5 | 10 | 17 | 6 | 5 | 8 | 20 | 15 | 15 | 25 | 25 | 5 |
|  | Y2 | 5 | 6 | 14 | 4 | 7 | 6 | 15 | 15 | 25 | 25 | 10 | 0 |
| Group 2 | Ea | 0 | 4 | 9 | 6 | 10 | 8 | 5 | 0 | 10 | 20 | 10 | 40 |
|  | Z1 | 0 | 4 | 12 | 6 | 3 | 2 | 5 | 0 | 0 | 0 | 10 | 30 |
|  | Z2 | 0 | 4 | 9 | 6 | 3 | 4 | 10 | 0 | 0 | 0 | 10 | 35 |
|  | T1 | 0 | 4 | 9 | 6 | 3 | 2 | 5 | 0 | 0 | 0 | 10 | 50 |
|  | T2 | 0 | 4 | 9 | 6 | 5 | 2 | 5 | 0 | 15 | 10 | 10 | 30 |

**Table F.** Values of topographic, geologic and land cover variables not included in Table 1. Note that the percentages do not have to sum 100%. Acronyms are shown in S1 Table.

|  |  | VWI | CALC | CONG | SDIM | PAS | BLF | CNF | PLT | SSH |
| --- | --- | --- | --- | --- | --- | --- | --- | --- | --- | --- |
| Group 1 | Enc | 08 | 08 | 91 | 00 | 07 | 21 | 00 | 03 | 22 |
|  | R1 | 28 | 14 | 86 | 00 | 02 | 24 | 14 | 35 | 24 |
|  | R2 | 25 | 11 | 89 | 00 | 02 | 22 | 11 | 27 | 20 |
|  | R3 | 10 | 05 | 94 | 02 | 02 | 21 | 05 | 13 | 10 |
|  | Y1 | 13 | 22 | 78 | 00 | 04 | 40 | 06 | 09 | 41 |
|  | Y2 | 44 | 09 | 90 | 01 | 04 | 50 | 06 | 05 | 27 |
| Group 2 | Ea | 33 | 10 | 87 | 03 | 02 | 02 | 01 | 00 | 15 |
|  | Z1 | 30 | 00 | 32 | 68 | 01 | 10 | 01 | 00 | 01 |
|  | Z2 | 20 | 00 | 33 | 67 | 01 | 10 | 01 | 00 | 01 |
|  | T1 | 50 | 00 | 97 | 03 | 02 | 17 | 03 | 03 | 01 |
|  | T2 | 26 | 00 | 89 | 11 | 06 | 08 | 02 | 02 | 01 |

**Table G.** Density (ind/m^2^) of macroinvertebrate taxa at each site.

|  | **Enc** | **R1** | **R2** | **R3** | **Y1** | **Y2** | **Ea** | **Z1** | **Z3** | **T1** | **T2** |
| --- | --- | --- | --- | --- | --- | --- | --- | --- | --- | --- | --- |
| *Baetis* | 1450.7 | 234.7 | 106.7 | 0.0 | 544.0 | 192.0 | 576.0 | 4896.0 | 2944.0 | 16320.0 | 6016.0 |
| *Caenis* | 2.0 | 0.0 | 0.0 | 16.0 | 0.0 | 0.0 | 32.0 | 96.0 | 64.0 | 0.0 | 0.0 |
| *Ephemerella* | 0.0 | 0.0 | 64.0 | 0.0 | 800.0 | 0.0 | 0.0 | 0.0 | 0.0 | 0.0 | 0.0 |
| *Ephemera* | 0.0 | 0.0 | 0.0 | 0.0 | 2.0 | 4.0 | 0.0 | 1.0 | 1.0 | 0.0 | 0.0 |
| *Ecdyonurus* | 42.7 | 0.0 | 704.0 | 16.0 | 32.0 | 16.0 | 0.0 | 0.0 | 0.0 | 0.0 | 0.0 |
| *Epeorus* | 0.0 | 320.0 | 0.0 | 0.0 | 64.0 | 32.0 | 0.0 | 0.0 | 0.0 | 0.0 | 0.0 |
| *Rithrogena* | 0.0 | 64.0 | 192.0 | 0.0 | 64.0 | 16.0 | 0.0 | 0.0 | 0.0 | 0.0 | 0.0 |
| *Habroleptoides* | 0.0 | 0.0 | 21.3 | 0.0 | 16.0 | 3.0 | 0.0 | 0.0 | 0.0 | 0.0 | 0.0 |
| *Habrophlebia* | 0.0 | 0.0 | 0.0 | 48.0 | 0.0 | 0.0 | 0.0 | 0.0 | 0.0 | 0.0 | 0.0 |
| *Paraleptophlebia* | 0.0 | 21.3 | 1.0 | 0.0 | 0.0 | 0.0 | 0.0 | 0.0 | 0.0 | 0.0 | 0.0 |
| *Cloroperla* | 0.0 | 2.0 | 0.0 | 0.0 | 1.0 | 0.0 | 0.0 | 0.0 | 0.0 | 0.0 | 0.0 |
| *Leuctra* | 21.3 | 21.3 | 85.3 | 0.0 | 768.0 | 32.0 | 0.0 | 0.0 | 0.0 | 0.0 | 0.0 |
| *Protonemura* | 0.0 | 1.0 | 0.0 | 0.0 | 0.0 | 0.0 | 0.0 | 0.0 | 0.0 | 0.0 | 0.0 |
| *Nemoura* | 0.0 | 0.0 | 0.0 | 3.0 | 1.0 | 0.0 | 0.0 | 0.0 | 1.0 | 0.0 | 0.0 |
| *Perla* | 0.0 | 1.0 | 0.0 | 0.0 | 64.0 | 0.0 | 0.0 | 0.0 | 0.0 | 0.0 | 0.0 |
| *Isoperla* | 0.0 | 0.0 | 7.0 | 1.0 | 1.0 | 3.0 | 0.0 | 0.0 | 0.0 | 0.0 | 0.0 |
| *Calopteryx* | 0.0 | 1.0 | 0.0 | 0.0 | 0.0 | 0.0 | 32.0 | 0.0 | 32.0 | 0.0 | 0.0 |
| *Coenagrion* | 0.0 | 0.0 | 0.0 | 1.0 | 0.0 | 0.0 | 0.0 | 0.0 | 3.0 | 0.0 | 2.0 |
| *Cordulegaster* | 1.0 | 0.0 | 0.0 | 3.0 | 0.0 | 0.0 | 0.0 | 0.0 | 0.0 | 0.0 | 0.0 |
| *Gomphus* | 0.0 | 0.0 | 0.0 | 0.0 | 1.0 | 0.0 | 0.0 | 0.0 | 0.0 | 0.0 | 0.0 |
| *Gerris* | 2.0 | 0.0 | 0.0 | 0.0 | 0.0 | 1.0 | 0.0 | 0.0 | 0.0 | 0.0 | 0.0 |
| *Nepa* | 0.0 | 0.0 | 0.0 | 0.0 | 1.0 | 0.0 | 0.0 | 0.0 | 0.0 | 0.0 | 0.0 |
| *Microvelia* | 0.0 | 21.3 | 0.0 | 0.0 | 0.0 | 0.0 | 0.0 | 0.0 | 1.0 | 0.0 | 0.0 |
| *Velia* | 0.0 | 3.0 | 0.0 | 1.0 | 0.0 | 0.0 | 0.0 | 0.0 | 0.0 | 0.0 | 0.0 |
| *Macroplea* | 0.0 | 0.0 | 0.0 | 1.0 | 0.0 | 0.0 | 0.0 | 0.0 | 0.0 | 0.0 | 0.0 |
| *Agabus* | 0.0 | 0.0 | 0.0 | 0.0 | 0.0 | 0.0 | 1.0 | 0.0 | 0.0 | 2.0 | 0.0 |
| *Dytiscus* | 1.0 | 0.0 | 0.0 | 1.0 | 0.0 | 0.0 | 0.0 | 0.0 | 0.0 | 0.0 | 0.0 |
|  | **Enc** | **R1** | **R2** | **R3** | **Y1** | **Y2** | **Ea** | **Z1** | **Z3** | **T1** | **T2** |
| *Laccophilus* | 0.0 | 0.0 | 0.0 | 1.0 | 0.0 | 0.0 | 0.0 | 0.0 | 0.0 | 0.0 | 0.0 |
| *Platambus* | 0.0 | 0.0 | 0.0 | 0.0 | 32.0 | 0.0 | 0.0 | 0.0 | 0.0 | 0.0 | 0.0 |
| *Scarodytes* | 0.0 | 0.0 | 0.0 | 0.0 | 16.0 | 0.0 | 0.0 | 0.0 | 0.0 | 0.0 | 0.0 |
| *Dupophilus* | 0.0 | 0.0 | 0.0 | 0.0 | 4.0 | 0.0 | 0.0 | 0.0 | 0.0 | 0.0 | 0.0 |
| *Elmis* | 256.0 | 128.0 | 192.0 | 80.0 | 192.0 | 128.0 | 368.0 | 0.0 | 0.0 | 0.0 | 0.0 |
| *Esolus* | 85.3 | 11.0 | 170.7 | 176.0 | 640.0 | 192.0 | 2.0 | 0.0 | 0.0 | 0.0 | 0.0 |
| *Limnius* | 405.3 | 6.0 | 4.0 | 240.0 | 64.0 | 6.0 | 1.0 | 32.0 | 64.0 | 0.0 | 0.0 |
| *Oulimnius* | 21.3 | 0.0 | 0.0 | 0.0 | 0.0 | 0.0 | 16.0 | 0.0 | 0.0 | 0.0 | 0.0 |
| *Stenelmis* | 0.0 | 0.0 | 0.0 | 0.0 | 0.0 | 0.0 | 0.0 | 0.0 | 0.0 | 0.0 | 0.0 |
| *Haliplus* | 4.0 | 0.0 | 0.0 | 3.0 | 5.0 | 0.0 | 80.0 | 1.0 | 1.0 | 128.0 | 64.0 |
| *Helophorus* | 7.0 | 21.3 | 1.0 | 5.0 | 0.0 | 0.0 | 2.0 | 0.0 | 0.0 | 0.0 | 2.0 |
| *Hidraena* | 21.3 | 31.3 | 1.0 | 0.0 | 128.0 | 112.0 | 1.0 | 0.0 | 0.0 | 0.0 | 1.0 |
| *Elodes* | 0.0 | 4.0 | 42.7 | 0.0 | 0.0 | 0.0 | 0.0 | 0.0 | 0.0 | 0.0 | 0.0 |
| *Hydrocyphon* | 0.0 | 0.0 | 0.0 | 0.0 | 128.0 | 0.0 | 0.0 | 0.0 | 0.0 | 0.0 | 0.0 |
| *Synagapetus* | 0.0 | 4.0 | 0.0 | 0.0 | 0.0 | 0.0 | 0.0 | 0.0 | 0.0 | 0.0 | 0.0 |
| *Hydropsyche* | 21.3 | 192.0 | 0.0 | 7.0 | 352.0 | 208.0 | 0.0 | 1.0 | 9.0 | 0.0 | 9.0 |
| *Hydroptila* | 0.0 | 0.0 | 0.0 | 0.0 | 0.0 | 0.0 | 176.0 | 192.0 | 32.0 | 1.0 | 0.0 |
| *Adicella* | 0.0 | 2.0 | 0.0 | 0.0 | 0.0 | 0.0 | 0.0 | 0.0 | 0.0 | 0.0 | 0.0 |
| Limnephilinae | 21.3 | 0.0 | 1.0 | 1.0 | 16.0 | 1.0 | 1.0 | 0.0 | 0.0 | 0.0 | 0.0 |
| *Odontocerum* | 0.0 | 0.0 | 0.0 | 0.0 | 32.0 | 0.0 | 0.0 | 0.0 | 0.0 | 0.0 | 0.0 |
| *Philopotamus* | 0.0 | 21.3 | 0.0 | 0.0 | 1.0 | 32.0 | 0.0 | 0.0 | 0.0 | 0.0 | 0.0 |
| *Chimarra* | 0.0 | 0.0 | 0.0 | 0.0 | 0.0 | 1.0 | 0.0 | 0.0 | 0.0 | 0.0 | 0.0 |
| *Polycentropus* | 0.0 | 0.0 | 0.0 | 0.0 | 1.0 | 0.0 | 0.0 | 0.0 | 0.0 | 0.0 | 0.0 |
| *Plectrocnemia* | 1.0 | 21.3 | 21.3 | 16.0 | 0.0 | 48.0 | 0.0 | 0.0 | 0.0 | 0.0 | 0.0 |
| *Lype* | 0.0 | 1.0 | 0.0 | 0.0 | 0.0 | 3.0 | 0.0 | 0.0 | 0.0 | 0.0 | 0.0 |
| *Hyperhyacophila* | 3.0 | 1.0 | 0.0 | 0.0 | 16.0 | 5.0 | 16.0 | 5.0 | 0.0 | 0.0 | 0.0 |
| *Sericostoma* | 0.0 | 7.0 | 0.0 | 0.0 | 5.0 | 0.0 | 0.0 | 0.0 | 0.0 | 0.0 | 0.0 |
| *Elophila* | 0.0 | 0.0 | 1.0 | 0.0 | 0.0 | 0.0 | 0.0 | 0.0 | 0.0 | 0.0 | 0.0 |
| Atrichops | 0.0 | 0.0 | 0.0 | 2.0 | 0.0 | 0.0 | 0.0 | 0.0 | 0.0 | 0.0 | 0.0 |
| Ceratopogoninae | 42.7 | 85.3 | 0.0 | 9.0 | 128.0 | 2.0 | 1.0 | 0.0 | 0.0 | 0.0 | 0.0 |
|  | **Enc** | **R1** | **R2** | **R3** | **Y1** | **Y2** | **Ea** | **Z1** | **Z3** | **T1** | **T2** |
| Orthocladiinae | 307.2 | 150.3 | 184.9 | 469.3 | 376.6 | 160.0 | 613.4 | 1379.8 | 1643.8 | 4356.0 | 2220.2 |
| Chironominae | 76.8 | 75.1 | 46.2 | 19.6 | 83.7 | 30.8 | 216.5 | 125.4 | 690.4 | 7524.0 | 3795.8 |
| Tanypodinae | 0.0 | 350.6 | 46.2 | 39.1 | 83.7 | 49.2 | 18.0 | 62.7 | 65.8 | 792.0 | 0.0 |
| Anophelinae | 0.0 | 0.0 | 0.0 | 0.0 | 0.0 | 0.0 | 1.0 | 0.0 | 0.0 | 0.0 | 0.0 |
| *Dixa* | 1.0 | 0.0 | 1.0 | 16.0 | 2.0 | 0.0 | 0.0 | 1.0 | 0.0 | 0.0 | 0.0 |
| Clinocerinae | 0.0 | 0.0 | 0.0 | 0.0 | 0.0 | 16.0 | 0.0 | 0.0 | 1.0 | 32.0 | 0.0 |
| Hemerodrominae | 0.0 | 0.0 | 0.0 | 0.0 | 16.0 | 0.0 | 0.0 | 0.0 | 0.0 | 0.0 | 0.0 |
| *Pediciini* | 3.0 | 1.0 | 0.0 | 0.0 | 5.0 | 0.0 | 16.0 | 0.0 | 0.0 | 0.0 | 0.0 |
| Psychodidae | 2.0 | 0.0 | 2.0 | 16.0 | 7.0 | 0.0 | 16.0 | 192.0 | 32.0 | 384.0 | 1.0 |
| Rhagionidae | 21.3 | 1.0 | 0.0 | 0.0 | 0.0 | 3.0 | 0.0 | 1.0 | 0.0 | 0.0 | 0.0 |
| Scatophagidae | 0.0 | 1.0 | 0.0 | 0.0 | 0.0 | 0.0 | 0.0 | 0.0 | 0.0 | 0.0 | 0.0 |
| Simuliini | 42.7 | 21.3 | 0.0 | 0.0 | 32.0 | 0.0 | 16.0 | 1376.0 | 64.0 | 256.0 | 864.0 |
| Stratiomyidae | 21.3 | 0.0 | 2.0 | 0.0 | 0.0 | 0.0 | 0.0 | 0.0 | 0.0 | 0.0 | 0.0 |
| Tipulidae | 0.0 | 0.0 | 0.0 | 0.0 | 0.0 | 2.0 | 2.0 | 5.0 | 2.0 | 2.0 | 0.0 |
| *Proasellus* | 1.0 | 0.0 | 2.0 | 0.0 | 0.0 | 0.0 | 16.0 | 0.0 | 0.0 | 0.0 | 0.0 |
| Gammaridae | 2752.0 | 3221.3 | 5952.0 | 3200.0 | 32.0 | 2304.0 | 0.0 | 32.0 | 128.0 | 1.0 | 0.0 |
| *Pacifastacus* | 0.0 | 0.0 | 0.0 | 0.0 | 0.0 | 0.0 | 2.0 | 0.0 | 0.0 | 0.0 | 0.0 |
| *Hydrachna* | 0.0 | 21.3 | 2.0 | 16.0 | 64.0 | 32.0 | 112.0 | 1.0 | 96.0 | 0.0 | 0.0 |
| *Ancylus fluviatilis* | 0.0 | 2.0 | 21.3 | 0.0 | 0.0 | 4.0 | 0.0 | 1.0 | 1.0 | 1.0 | 0.0 |
| *Bythiospeum* | 0.0 | 0.0 | 277.3 | 16.0 | 0.0 | 0.0 | 768.0 | 0.0 | 0.0 | 0.0 | 0.0 |
| *Radix* | 0.0 | 0.0 | 0.0 | 0.0 | 0.0 | 16.0 | 0.0 | 0.0 | 0.0 | 0.0 | 0.0 |
| *Physa* | 0.0 | 0.0 | 0.0 | 0.0 | 0.0 | 0.0 | 1.0 | 0.0 | 0.0 | 0.0 | 1.0 |
| *Sphaerium* | 0.0 | 1.0 | 0.0 | 0.0 | 0.0 | 1.0 | 32.0 | 0.0 | 2.0 | 0.0 | 32.0 |
| *Valvata* | 0.0 | 0.0 | 0.0 | 0.0 | 0.0 | 16.0 | 0.0 | 0.0 | 0.0 | 0.0 | 0.0 |
| *Erpobdella* | 3.0 | 2.0 | 0.0 | 2.0 | 4.0 | 0.0 | 0.0 | 8.0 | 1.0 | 160.0 | 32.0 |
| *Glossiphonia* | 0.0 | 0.0 | 0.0 | 0.0 | 1.0 | 0.0 | 0.0 | 0.0 | 0.0 | 4.0 | 0.0 |
| *Helobdella stagnalis* | 0.0 | 0.0 | 0.0 | 0.0 | 0.0 | 0.0 | 0.0 | 0.0 | 0.0 | 0.0 | 1.0 |
| *Polycelis* | 0.0 | 21.3 | 170.7 | 1.0 | 0.0 | 16.0 | 0.0 | 0.0 | 0.0 | 0.0 | 0.0 |
| Oligochaeta | 85.3 | 256.0 | 1.0 | 512.0 | 256.0 | 32.0 | 416.0 | 192.0 | 1120.0 | 17856.0 | 1984.0 |

**Table H.** Relative abundance of each macroinvertebrate functional trait at study sites.

| **Biological trait** | **Category** | **Enc** | **R1** | **R2** | **R3** | **Y1** | **Y2** | **Ea** | **Z1** | **Z2** | **T1** | **T2** |
| --- | --- | --- | --- | --- | --- | --- | --- | --- | --- | --- | --- | --- |
| Reproductive cycles | less than 1 | 0.080 | 0.073 | 0.092 | 0.087 | 0.109 | 0.113 | 0.030 | 0.053 | 0.063 | 0.058 | 0.019 |
|  | 1 | 0.559 | 0.611 | 0.693 | 0.573 | 0.622 | 0.622 | 0.544 | 0.411 | 0.407 | 0.402 | 0.372 |
|  | more than 1 | 0.361 | 0.316 | 0.215 | 0.340 | 0.269 | 0.266 | 0.426 | 0.536 | 0.530 | 0.539 | 0.609 |
| Respiration | tegument | 0.340 | 0.445 | 0.438 | 0.388 | 0.387 | 0.501 | 0.409 | 0.498 | 0.500 | 0.645 | 0.596 |
|  | gill | 0.432 | 0.392 | 0.465 | 0.404 | 0.420 | 0.386 | 0.392 | 0.332 | 0.379 | 0.192 | 0.274 |
|  | plastron | 0.117 | 0.063 | 0.065 | 0.089 | 0.076 | 0.082 | 0.061 | 0.022 | 0.025 | 0.000 | 0.012 |
|  | spiracle | 0.111 | 0.100 | 0.032 | 0.120 | 0.117 | 0.032 | 0.139 | 0.149 | 0.096 | 0.163 | 0.118 |
|  | hydrostatic vesicle | 0.000 | 0.000 | 0.000 | 0.000 | 0.000 | 0.000 | 0.000 | 0.000 | 0.000 | 0.000 | 0.000 |
| Locomotion | flier | 0.052 | 0.031 | 0.024 | 0.041 | 0.035 | 0.030 | 0.032 | 0.010 | 0.013 | 0.011 | 0.015 |
|  | surface swimmer | 0.020 | 0.042 | 0.016 | 0.041 | 0.013 | 0.017 | 0.021 | 0.030 | 0.024 | 0.027 | 0.008 |
|  | full water swimmer | 0.155 | 0.154 | 0.123 | 0.155 | 0.143 | 0.131 | 0.146 | 0.156 | 0.162 | 0.215 | 0.198 |
|  | crawler | 0.504 | 0.476 | 0.587 | 0.474 | 0.561 | 0.508 | 0.461 | 0.447 | 0.450 | 0.387 | 0.353 |
|  | burrower | 0.077 | 0.081 | 0.048 | 0.085 | 0.096 | 0.093 | 0.106 | 0.101 | 0.107 | 0.109 | 0.125 |
|  | interstitial | 0.117 | 0.100 | 0.149 | 0.138 | 0.086 | 0.104 | 0.159 | 0.127 | 0.135 | 0.144 | 0.130 |
|  | temporarily attached | 0.075 | 0.113 | 0.053 | 0.065 | 0.066 | 0.116 | 0.075 | 0.129 | 0.109 | 0.108 | 0.171 |
|  | permanently attached | 0.000 | 0.001 | 0.000 | 0.000 | 0.000 | 0.002 | 0.000 | 0.000 | 0.000 | 0.000 | 0.000 |
| Food | microorg. + fine sediment | 0.019 | 0.012 | 0.017 | 0.025 | 0.016 | 0.017 | 0.021 | 0.025 | 0.031 | 0.037 | 0.034 |
|  | detritus < 1mm | 0.221 | 0.199 | 0.175 | 0.200 | 0.180 | 0.189 | 0.207 | 0.272 | 0.257 | 0.232 | 0.287 |
|  | dead plant >= 1mm | 0.098 | 0.074 | 0.137 | 0.116 | 0.084 | 0.099 | 0.104 | 0.105 | 0.099 | 0.082 | 0.047 |
|  | living microphytes | 0.383 | 0.349 | 0.363 | 0.346 | 0.356 | 0.359 | 0.331 | 0.282 | 0.284 | 0.279 | 0.350 |
|  | living macrophytes | 0.058 | 0.065 | 0.099 | 0.053 | 0.079 | 0.067 | 0.136 | 0.103 | 0.085 | 0.089 | 0.090 |
|  | dead animal >= 1mm | 0.038 | 0.044 | 0.049 | 0.041 | 0.027 | 0.036 | 0.030 | 0.053 | 0.042 | 0.058 | 0.037 |
|  | living microinvertebrates | 0.064 | 0.105 | 0.050 | 0.078 | 0.091 | 0.082 | 0.047 | 0.075 | 0.084 | 0.081 | 0.085 |
|  | living macroinvertebrates | 0.115 | 0.152 | 0.110 | 0.137 | 0.159 | 0.150 | 0.122 | 0.084 | 0.118 | 0.138 | 0.067 |
|  | vertebrates | 0.004 | 0.000 | 0.000 | 0.004 | 0.008 | 0.000 | 0.002 | 0.000 | 0.000 | 0.003 | 0.003 |
| **Biological trait** | **Category** | **Enc** | **R1** | **R2** | **R3** | **Y1** | **Y2** | **Ea** | **Z1** | **Z2** | **T1** | **T2** |
| Feeding habits | absorber | 0.000 | 0.000 | 0.000 | 0.000 | 0.000 | 0.000 | 0.000 | 0.000 | 0.000 | 0.000 | 0.000 |
|  | deposit feeder | 0.113 | 0.121 | 0.081 | 0.138 | 0.105 | 0.096 | 0.164 | 0.225 | 0.224 | 0.231 | 0.211 |
|  | shredder | 0.258 | 0.184 | 0.289 | 0.297 | 0.258 | 0.215 | 0.187 | 0.163 | 0.172 | 0.147 | 0.085 |
|  | scraper | 0.375 | 0.346 | 0.437 | 0.282 | 0.337 | 0.383 | 0.308 | 0.279 | 0.267 | 0.259 | 0.302 |
|  | filter-feeder | 0.082 | 0.102 | 0.042 | 0.075 | 0.074 | 0.116 | 0.104 | 0.125 | 0.119 | 0.095 | 0.225 |
|  | piercer | 0.056 | 0.038 | 0.000 | 0.037 | 0.037 | 0.019 | 0.060 | 0.053 | 0.036 | 0.061 | 0.048 |
|  | predator | 0.102 | 0.200 | 0.140 | 0.156 | 0.178 | 0.160 | 0.162 | 0.135 | 0.159 | 0.177 | 0.098 |
|  | parasite | 0.013 | 0.010 | 0.012 | 0.015 | 0.010 | 0.011 | 0.016 | 0.021 | 0.022 | 0.031 | 0.030 |
| Microhabitat | boulders/cobbles/pebbles | 0.219 | 0.290 | 0.241 | 0.170 | 0.253 | 0.293 | 0.165 | 0.204 | 0.175 | 0.185 | 0.197 |
|  | gravel | 0.145 | 0.124 | 0.152 | 0.124 | 0.147 | 0.137 | 0.123 | 0.115 | 0.121 | 0.121 | 0.103 |
|  | sand | 0.117 | 0.092 | 0.119 | 0.142 | 0.112 | 0.100 | 0.130 | 0.097 | 0.106 | 0.096 | 0.098 |
|  | silt | 0.059 | 0.037 | 0.083 | 0.095 | 0.057 | 0.045 | 0.107 | 0.052 | 0.062 | 0.068 | 0.063 |
|  | macrophytes | 0.220 | 0.198 | 0.175 | 0.193 | 0.193 | 0.189 | 0.218 | 0.234 | 0.234 | 0.229 | 0.246 |
|  | microphytes | 0.036 | 0.039 | 0.029 | 0.033 | 0.022 | 0.028 | 0.042 | 0.032 | 0.032 | 0.025 | 0.037 |
|  | twigs/roots | 0.077 | 0.088 | 0.091 | 0.066 | 0.089 | 0.090 | 0.062 | 0.095 | 0.096 | 0.078 | 0.084 |
|  | organic detritus/litter | 0.077 | 0.076 | 0.077 | 0.098 | 0.073 | 0.064 | 0.069 | 0.089 | 0.092 | 0.094 | 0.077 |
|  | mud | 0.049 | 0.056 | 0.033 | 0.080 | 0.054 | 0.053 | 0.085 | 0.081 | 0.083 | 0.102 | 0.095 |
| Trophic status | oligotrophic | 0.426 | 0.450 | 0.514 | 0.437 | 0.448 | 0.469 | 0.400 | 0.317 | 0.308 | 0.254 | 0.232 |
|  | mesotrophic | 0.401 | 0.372 | 0.389 | 0.393 | 0.402 | 0.389 | 0.392 | 0.429 | 0.444 | 0.438 | 0.466 |
|  | eutrophic | 0.173 | 0.178 | 0.098 | 0.170 | 0.150 | 0.142 | 0.208 | 0.255 | 0.248 | 0.308 | 0.302 |
