# Supplementary material for: Agricultural impacts on streams near Nitrate Vulnerable Zones: A case study in the Ebro basin, Northern Spain
Source: PLoS One. 2019 Nov 8;14(11):e0218582. doi: 10.1371/journal.pone.0218582 (PMC6839874; doi:10.1371/journal.pone.0218582)
Supplement: Attachment — Submitted filename: Response to reviewers_final.docx [file pone.0218582.s002.docx]

Dear Editor,

First of all, we would like to thank you and the reviewers for the valuable contributions to the manuscript, which have considerably improved it. We have included new tables in the Supporting Information and modified tables and figures in the manuscript according to the reviewer´s comments. We have also modified the structure of the different sections (i.e. rearranged them) and the content of the manuscript (e.g. by providing additional information to clarify some issues).

Below we provide detailed answers to every major comment and to minor ones when necessary (some, such as minor text modifications, have been incorporated without further discussion).

**Reviewer 1**

**Major comments**

**1. Reviewer comment: Regarding the use of Mann-Whitney U test for the analysis of trait composition. Although it is a non-parametric test, Mann-Whitney U test assumes that the distributions are similar. Figure 4 shows that in several cases the distributions of the two groups are not similar. I recommend choosing a more appropriate analysis. An alternative approach could be applying IndVal analysis to trait compositional data.**

Response: Mann-Whitney U test assumes that, under the null hypothesis H0, the distributions of both groups are equal, and under the alternative hypothesis H1 the distributions are not equal. Therefore, we consider that this test is appropriate to determine functional differences between groups. Nevertheless, following the reviewer’s suggestion, we have performed an IndVal analysis. The results are shown below:

| Trait category | group | indval | pvalue | Trait category | group | indval | pvalue |
| --- | --- | --- | --- | --- | --- | --- | --- |
| Fedding habits: deposit_feeder | 2 | 0,659 | 0,002 | Microhabitat: bould_cobbl_peb | 1 | 0,569 | 0,029 |
| Fedding habits: filter_feeder | 2 | 0,620 | 0,028 | Microhabitat: gravels | 1 | 0,542 | 0,014 |
| Fedding habits: parasite | 2 | 0,673 | 0,002 | Microhabitat: macrophytes | 2 | 0,588 | 0,018 |
| Fedding habits: scraper | 1 | 0,560 | 0,012 | Microhabitat: mud | 2 | 0,622 | 0,004 |
| Fedding habits: shredder | 1 | 0,624 | 0,008 | Reprod. Cycles: 1 | 1 | 0,589 | 0,004 |
| Food: detritus_less1 | 2 | 0,564 | 0,007 | Reprod. Cycles: less_1 | 1 | 0,674 | 0,006 |
| Food: fine_sediment | 2 | 0,627 | 0,008 | Reprod. Cycles: more_1 | 2 | 0,642 | 0,003 |
| Food: macrophytes | 2 | 0,544 | 0,006 | Respiration: gill | 1 | 0,570 | 0,009 |
| Food: microphytes | 1 | 0,541 | 0,005 | Respiration: plastron | 1 | 0,775 | 0,002 |
| Locomotion: burrower | 2 | 0,578 | 0,008 | Respiration: tegument | 2 | 0,560 | 0,037 |
| Locomotion: crawler | 1 | 0,553 | 0,003 | Trophic status: eutrophic | 2 | 0,635 | 0,003 |
| Locomotion: flier | 1 | 0,688 | 0,011 | Trophic status: mesotrophic | 2 | 0,526 | 0,009 |
| Locomotion: swimmer | 2 | 0,550 | 0,039 | Trophic status: oligotrophic | 1 | 0,602 | 0,003 |

A total of 26 functional traits were significantly associated to one of the two groups, 25 of which were also significantly different between group 1 and 2 according to the Mann-Whitney U test. Tegument respiration was the only trait selected by the IndVal analysis that did not differ significantly between groups according to the Mann-Whitney U test.

Since the results from the two analyses are very similar, we have decided to keep the Mann-Whitney U test in the manuscript. With only two groups, we were not interested in assigning an indicator value to each trait characteristic. Besides, as the groups were defined using macroinvertebrate composition, we consider that applying the Indval again on trait characteristics (as we have done with composition) could be redundant. Moreover, we think that the “beanplots” are very illustrative of the differences in trait characteristics (supported by the Mann-Whitney tests).

**2. Reviewer comment: It is not written if the authors tested the explanatory variables for collinearity before running the DISTLM analysis, which is an important step for this kind of analyses.**

Response: We have explained the DISTLM method in greater detail in the new version of the manuscript, including aspects related to the collinearity of variables. Variables highly correlated with others (i.e. Spearman correlation coefficient higher than 0.9) had been previously removed from the analysis.

**3. Reviewer comment: The authors state that “All relevant data are within the manuscript and its Supporting Information files”, but I could not find the taxonomic and trait composition of benthic assemblages at the studied sites in the current submission.**

Response: The taxonomic and trait composition of the macroinvertebrate assemblages at the studied sites have been included in the Supporting Information.

**Minor comments**

**1. Reviewer comment: How was the impact of urban/industrial/other anthropogenic pressures assessed? Did the assessment account for the area adjacent the stream or for the whole upstream catchment? What were the criteria for site selection? What was the size of the studied sites?**

Response: As we have detailed in the revised manuscript, to reduce natural differences among the studied sites (100 m length), all of them belong to first-order streams and to the same stream typology ("Calcareous Mediterranean mountain rivers") according to the classification developed in Spain by the Ministry for the Environment to fulfil the WFD (ORDER ARM/2656/2008). The study sites were not affected by urban or industrial discharges. There are no large villages (i.e. more than 500 inhabitants) in the area and almost no industrial activity (agriculture is the main activity). The study sites were selected to fulfil the above-mentioned criteria after a visual inspection of the region.

**Reviewer 2**

**Major comments**

**1. Reviewer comment: Some interesting data are presented which show high levels of pollution, particularly for the upstream areas of catchments. Indeed, it is concluded in lines 408-409 that NVZs and the WFD are not having an environmental impact, as has generally been found in the available literature, and this is a very important point. The authors need to state more clearly what is already known about the effectiveness of NVZs and include papers such as Howden *et al.* (2011), Kay *et al.* (2012), Weatherhead and Howden (2009), and Worrall *et al.* (2009).**

Response: We thank the reviewer for the recommended literature. We have revised it and included it in the “conclusion and recommendation” section to provide more information on the Nitrate Directive and the NVZs effectiveness, and to expand on the management implications of our study.

**Reviewer 3**

**Major comments**

**1. Reviewer comment: The conclusion that habitat degradation (e.g., sedimentation) is an important stress pathway from agricultural pressures rests largely on the results of the dbRDA and the trait analyses. However, I would like to see an explicit test of the key a priori hypothesis: namely, that nitrate is a major stress pathway in these streams (after all, nitrate is mentioned in the title). To do this explicitly, I would consider taking the constrained ordination further with variation partitioning to test if nitrate has a significant independent influence on macroinvertebrate community structure (see “varpart” in the “vegan” R package).**

Response: We have revised the text to better explain the use of DISTLM to determine the importance of the different environmental variables in the variation of the macroinvertebrate assemblages among sites. “Varpart” (vegan) partitions variance using a Redundancy Analysis, whereas DISTLM does it using a Distance-based Redundancy Analysis. The DISTLM analysis allows analysing and modelling the relationship between a biological data cloud (i.e. a composition resemblance matrix) and one or more predictor variables. In our case, the resemblance matrix described the dissimilarities among the macroinvertebrate assemblages of the studied sites based on genus abundance data, and we used the environmental variables (including nitrate) as predictor variables. The DISTLM provided quantitative measures of the variation explained by each predictor, allowing the partitioning of variance according to a regression model.

As it is described in the new version of the manuscript, according to DISTLM analysis, nitrate significantly explained a 9.3% of the variation in the macroinvertebrate assemblages between sites. However, the variation in aquatic macroinvertebrate assemblages among sites was best explained by the variables associated with hydromorphological alterations of the stream habitat and topography. Specifically, the combination of the IHF index, the QBR components that refer to riparian vegetation cover and river channel alteration, and the ratio of valley width to channel width (VWI) explained a 67% of the variation in the macroinvertebrate assemblage among sites.

**2.** **Reviewer comment: It would be good if the authors could highlight which of their environmental factors measured deposited sediment (i.e., yes, the IHF index tackles this but how?). At the moment, it is sort of inferred but not really explicit, which weakens the discussion.**

Response: Deposited sediment is directly related to the component 1 of the IHF index. As we have detailed in the new version of the manuscript and in the Supporting Information, this component refers to hard substrate embeddedness into the fine sediment. Table S4 shows that every site in group 2 has a score of 0 in this component, which means that more than 60% of the hard substrate was embedded in fine sediment, whereas every site of group 1 showed a higher score (i.e. lower embeddedness).

**3. Reviewer comment: There are other stressors and pressures that could contribute to changes that are not mentioned. Did the authors consider using the SPEAR index to assess potential pesticide impacts? Are there wastewater outfalls in the study basin that could contribute to the observed water quality issues? For example, site Z2 looks like it is downstream of a WWTP.**

Response: Regarding other stressors (e.g. pesticides) and indices (e.g. SPEAR) not included in the study, we would like to highlight that the Ebro Hydrographic Confederation (the public organism responsible of water quality monitoring programmes in the rivers of the area; http://www.chebro.es/) routinely analyses pesticides (together with nutrients and other chemical compounds) in the study region. Among them, nitrate has been historically pointed out as the main pollutant and stressor of these water bodies (public data on http://www.chebro.es/). Thus, although we recognise that it could be interesting to include pesticides in our study, we decided to focus on nitrate. Along with nutrients, we decided to assess hydromorphological variables because they have been pointed out as key factors shaping aquatic invertebrate communities in agricultural streams by previous papers and they have received little attention from the monitoring programmes.

Regarding wastewater treatment plants, we selected sites with no urban or industrial sewage discharges in their adjacent area. It should also be noted that there are no large villages (i.e. more than 500 inhabitants) in the area and almost no industrial activity (agriculture is the main activity). Z2 (belonging to Zamaca stream) is the closest site to a wastewater treatment plant, and it is located more than 2 km upstream of the sample site. Moreover, the wastewater plant is dimensioned to 3500 inhabitants (https://www.larioja.org/larioja-client/cm/consorcio-aguas/images?idMmedia=892956), and it collects discharges from 3 villages (Zarratón, Ollauri and Gimileo) that have 660 inhabitants in total (data from Spanish National Statistics Institute; INE). Thus, it guarantees an important reduction of pollutants before the effluent reaches Zamaca stream. For example, in 2017 nitrate concentration in the effluent of this wastewater plant was always lower than 18 ppm (https://www.larioja.org/consorcio-aguas/es/depuracion/instalaciones/depuradoras-servicio/estaciones-depuradoras-aguas-residuales), whereas the average nitrate concentration at Z2 was 84 ppm. Thus, the influence of the wastewater treatment plant seems to be very limited.

**4. Reviewer comment: Pressure vs stressors: these terms are used interchangeably in the manuscript which I believe detracts from the message. If you refer to the Driver, Pressure, State, Impact, and Response (DPSIR) framework, “pressures” refer to broad categories contributing to impairment (i.e., land-use change, pollution, etc.). The multiple-stressor literature on agricultural systems then treats different pollutants as “stressors”, e.g., nutrients (nitrate), sediment etc. Thus, agriculture could be conceived as the pressure, with the key stressors as identified.**

Response: The terms pressures and stressors have been corrected following the reviewer´s suggestions.

**Minor comments**

**1. Reviewer comment: Table 1. The sites could be ordered by their groups (i.e., 1 or 2). Are those nitrate concentrations for real!? Wow. Did the authors measure salinity too – the conductivities are extremely high too?**

Response: Following the reviewer’s suggestion, the sites have been ordered by group in the tables of the manuscript and in the Supporting Information. Relative to high conductivity values, as we have added to the manuscript, the high conductivity and concentrations of different ions at Enc and R3 sites are associated with natural sodium sulfate tertiary deposits and secondary minerals, mainly gypsum and calcite (Menduiña *et al.* 1984)

Menduiña J, Ordóñez S, del Cura MG. Geología del yacimiento de glauberita de Cerezo de Rio Tirón (Burgos). Bol Geol Min. 1984;95(1):35–51.

**Reviewer 4**

**Major comments**

**1. Reviewer comment: The introduction reads quite well: the objectives and the hypotheses that are going to be tested are clearly stated. However, several topics of prime importance – in my opinion – were eluded such as the complementarity between taxonomic and functional information, the importance of traits, the reasons why taxonomic composition is so used, etc. (see Truchy et al, Adv. Ecol Res 53, 55-96 for an overview and trait classification). Furthermore, I felt that the importance of the research question addressed here was lifted too late in the introduction (in the second to last paragraph) and that the transitions between the different paragraphs/ideas were abrupt.**

**L60-71: too many “the most”. This paragraph is very long and could easily get shortened for a more efficient introduction.**

Response: We thank the reviewer for the valuable suggestion and reference. We have rewritten the introduction, reduced the first paragraph and explained the concept of biological traits and its use in biomonitoring.

**2. Reviewer comment: The method section needs clearer justification for such sampling design (i.e. several sampling sites per stream, meaning the downstream sites are potentially capturing the effects already captured by the most upstream sites…). Also I have some concerns regarding the trait classification used by the authors. Although the authors seem to comply with the classification developed by Usseglio-Polatera *et al.*, 2000 (Freshwater Biol. 43, 175-205) that separates traits into biological vs. ecological traits, some of the traits used in this study are designated as biological traits but fall in reality in the ecological trait category (e.g. trophic status). I would rather recommend the authors to use another approach and consider response vs. effect traits as I think the hypotheses and therefore the conclusion of the study could be broaden to ecosystem functioning. In this respect, using traits belonging to the category of effect traits would definitely strengthen the message of the study. And least but not last, I think some complementary analyses are needed (maybe those were already conducted but then they should have been in the supplementary information). I am not very familiar with the statistical approach used by the authors (i.e. DISTLM) and therefore some justification regarding the choice of such statistics rather than the more “standard” set of analysis would have been appreciated (see my specific comments). I would also have liked to see 1) a PCA on the environmental variables rather than this very long and wordy description of the environmental characteristics of the sites in the result section; 2) a multivariate analysis on the species traits to observe the results in a multidimentional space and see which environmental variables trigger which trait (e.g. PCA, see Frainer *et al.* 2018, JAE 55: 377-385 for examples of analyses). What does DISTLM stand for? And what does it do? I guess the standard procedure would have been an NMDS followed by a PERMANOVA potentially complemented with a BEST procedure.**

Response: The sampling design has been explained and justified in the methods section of the manuscript. The 6 streams selected in the present study are the only first-order streams around these NVZs, where nitrate levels have previously exceeded, according to monitoring programs carried out by the Ebro Hydrographic Confederation (CHE; http://www.chebro.es/), the highest value established by WFD to consider that a river is in good ecological status. However, some of these streams have been studied just sporadically and all of them just at one site. Since the intensity of agricultural stressors may vary at reach scale, we included additional sampling sites.

We have now divided the functional traits into biological and ecological ones in accordance to Usseglio-Polatera *et al*., 2000, explaining the meaning of each one in the methodology and modifying Figure 4. We believe that this approach is suitable for our study, but we have also made a brief reference to the concept of effect traits both in methodology and in discussion. Finally, we have justified our selection of traits.

Usseglio-Polatera P, Bournaud M, Richoux P, Tachet H. Biological and ecological traits of benthic freshwater macroinvertebrates: Relationships and definition of groups with similar traits. Freshw Biol. 2000;43(2):175–205.

According to the reviewer’s comment, DISTLM analysis routine and its dbRDA associated plot have been better explained in the new version of the manuscript. We believe that no further analyses are required to meet our objectives, since the current analyses already quantify the response of the macroinvertebrate communities to shifts in the different predictor variables and identify the main traits that are affected by agricultural impacts. According to the manual of the PERMANOVA+ software, DISTLM is a more precise analogue of multiple linear regression than BEST, as it uses the resemblance matrix as an active sheet.

Frainier *et al.* (2018) used a PCA to summarise the variables in components that were then used in further analyses. This is an appropriate procedure to summarize information when the use of too many variables can difficult the observation of patterns. However, the observed relationships between two specific variables depend largely on the set of variables (adding or deleting variables may alter the apparent relationships among them) and the number of dimensions considered to represent the results. As we were not interested in summarising variables into components, we used the DISTLM to observe relationships between biological and environmental variables (without the influence of the set of variables or number of dimensions considered). The differences in functional characteristics are determined by the differences in composition between the two groups of sites defined, which are shaped by the different environmental conditions. These functional differences are illustrated using beanplots (statistically tested using Mann-Whitney).

**3. Reviewer comment: For the result section, the first section could be easily cleared up by removing all the unnecessary acronyms and site names and focus only on the environmental variables that the authors do discuss later. Also, a multivariate analysis on those environmental data would actually be an efficient way to summarize the information. Also, there is still some confusion around the DISTLM analysis as the authors seem to present a dbRDA in Figure 3. Finally, I would also suggest the authors to run a multivariate analysis on the trait data as already mentioned.**

Response: According to the reviewer’s comments, we have synthesized the environmental variables, focusing on those that are later discussed. Also, we have grouped the sites in the tables according to the cluster analysis to make the results easier to follow. We consider necessary to include these tables, since they show the physicochemical characterisation of the sites and their ecological status according to the WFD, which was one of the objectives of the study. Regarding the PCA analysis, please see our reply above.

**4.** **Reviewer comment: The discussion is overall well written and broaden quite nicely on recommendations for managers. But it would gain in strength if the authors started by making a “summary” paragraph where they would highlight their main results and whether or not their initial hypotheses were supported. By using response vs. effect traits, the authors could also extrapolate their results to stream ecosystem functioning, something that they just touch upon. This would definitively add some interest to the discussion.**

Response: According to the reviewer’s comments, we have added a “summary” paragraph to the discussion section where the main results and their relationship with our initial hypotheses are summarised. We have also made a brief reference to the concept of effect traits both in methodology and in discussion

**Minor comments**

**1. Reviewer comment: Figure 4, it is very hard to distinguish the white lines unfortunately. I would also recommend the authors to draw asterisks when the differences between the means are significant.**

Response: Figure 4 has been modified according to the trait classification -biological or ecological- and it has been redrawn to make lines more visible. We did not draw asterisks as the figure only includes trait categories that were significantly different between groups (this is explained in the figure caption).

**Reviewer 5**

**Major comments**

**1. Reviewer comment: The conclusions and recommendations are far outside the scope of, and thus unsupported by, the evidence presented here. Please constrain the conclusions and recommendations to those justifiable directly from the evidence. Your data and analyses do not address the efficacy of policies or practices (e.g., “dredging and channelization restrictions”) in achieving a reduction in the deleterious factors you have identified; it is possible there is are better strategies to achieve the desired beneficial outcome. Your data can only support the conclusion that less habitat alteration may improve ecological status.**

We have reworded the conclusions and recommendations to avoid overstatements and stick to the evidences supported by our results. Those addressing the efficacy of policies or practices in achieving a reduction in environmental stressors have been removed.

**2. Reviewer comment: How was macroinvertebrate sampling effort (to include enumeration effort) standardized across samples? Were adjustments made during statistical analysis where appropriate to account for any influence of variable sample effort? The macroinvertebrate enumeration protocol described in the manuscript appears to allow for a variable level of effort in enumeration (I trust that sample collection effort per Spanish protocol is standardized among sites; if not, that is of concern as well and should be addressed). As written, the manuscript relates an unclear and potentially inconsistent sub-sampling approach that could yield samples with vastly different number of individuals (level of effort) per sample. As written, the text suggests that some samples may have been sub-sampled, some may not have been, and the criteria are not clearly presented that were used for deciding when to subsample and when to stop counting after 300 individuals. As greater effort would naturally yield greater taxa richness (with implications for statistical analysis and interpretation), it is important to ensure that any observed increase in richness is not an artifact of the sampling procedure.**

The macroinvertebrate sampling and identification protocol has been described in the new version of the manuscript according to the reviewer’s comments. This quantitative protocol considers the presence of 5 different habitat types: hard substrate, fine sediments, plant debris, submerged bank vegetation and submerged macrophytes. Then, 20 surber samples (0.1 m^2^ surface) are taken, proportionally distributed among each type of habitat according to the percentage of streambed surface occupied by each of them. For counting macroinvertebrates, sub-sampling was used to estimate the taxa abundances once at least 300 individuals per sample were counted, although sample exploration continued to check for new taxa.

**3. Reviewer comment: Results section could be improved to better convey synthesized findings, as opposed to summary of measurements (which are in the Tables). By that I mean, tell the reader what you found; what should the reader take away after reading Results? To me, this section would be more compelling if it focused on conveying the findings that certain abiotic factors differed between biologically-different groups 1 and 2.**

**Additionally, the section could benefit from a bit more context; frame values relative to what is expected in streams of the region with minimal impact so the reader understands whether those values are of concern.**

**As well, or at the least, I suggest rearranging all presentation of data in tables, and possibly in text, to be grouped by the site groupings that were developed through cluster analysis (as opposed to the alphabetical arrangement at present). As those groupings are used for Fig 3 and Fig 4, seeing the abiotic data in tables grouped accordingly should facilitate easier contemplation of associations between abiotic and biotic data. For example, it is notable that, with a few exceptions, sites in Group 1 have lower specific conductance than sites in Group 2. Such an adjustment could also work well to more clearly convey differences between groups as presented in Results L211-261 and make the entire section more compelling. Given that you have established that biological differences exist between groups (Fig 2, Fig 3), presenting the abiotic information accordingly is more ecologically meaningful than site abbreviations that mean nothing to the unacquainted reader.**

We have synthesized the results section and focused on findings that were relevant for the objectives and discussion of the paper. Standards for the environmental variables and biotic indices included in the Spanish legislation (RD 817/2015), based on WFD, are shown in Table 1, as well as the ranges of the IHF and QBR indices. We have also rearranged data presentation (e.g. grouping sites according to groups obtained in cluster analysis and focusing on differences between established groups).

**4. Reviewer comment: Regarding averaging physicochemical parameters (L191-193, L225, and Tables 1 and S3), I generally agree that an annual mean derived from quarterly sampling can give a better long-term or overall representation of conditions to which organisms are exposed as compared to just one season of data. However, such averaging can mask seasonally-variable parameters that may have different effects at different seasons.**

**Summer, for example, is a particularly stressful time of year as it combines heat stress (summer maximum temperature) with low dissolved oxygen saturation potential (resulting from said heat). Similarly, nutrient levels are potentially of greater influence during the growing season (or leaf-off season) when sunlight and/or temperature are at levels sufficient to induce rapid excessive algal growth. Further, presenting the standard error of the mean in Table 1 is of marginal utility in describing values that vary widely across seasons (as noted with nitrate on L236-237). I’d prefer to see mean and range, the latter of which is helpful for understanding if extreme values (e.g., low summer oxygen, high summer temperature) may be influencing biota.**

According to reviewer´s suggestion, we show the mean and the range (i.e. minimum and maximum) of the physicochemical variables.

**Minor comments**

**1. Reviewer comment: On what basis has sedimentation been parsed from other factors in this study as a main driver of degradation? The largest proportion of variation was noted as the aggregate IHF-total, and no sediment-specific parameters are included in the list of other significant explanatory factors (L270-279). Presumably sediment components of the IHF were included in DISTLM just as was done with QBR-natur, yet those IHF components were not prominent factors differentiating groups. I don’t think the data and analyses here support such a narrow conclusion as written.**

We considered sedimentation as the main driver of degradation in sites affected by intensive agriculture because of two main issues:

a. The IHF index explained the largest proportion of variation among sites according to the DISTLM analysis. The first component of this index (IHF-1) refers to hard substrate embeddedness in fine sediment, although it resulted significantly correlated with the IHF total index (i.e. Spearman correlation coefficient higher than 0.9) and it was consequently removed from the DISTLM analysis. Anyway, as it can be seen in table S4, every site of group 2 has a score of 0 in this IHF component, which means that more than 60% of hard substrate was embedded in fine sediment; whereas every site of group 1 showed lower embeddedness and, consequently a higher score of this component.
b. Moreover, as we now state in the discussion, several traits significantly associated with the sites more impacted by agriculture seemed to be related to sediment increase. Accordingly, Murphy *et al*. (2017) confirmed the potential of macroinvertebrate biological traits as indicators of fine sediment impacts on rivers.

Murphy JF, Jones JI, Arnold A, Duerdoth CP, Pretty JL, Naden PS, *et al.* Can macroinvertebrate biological traits indicate fine-grained sediment conditions in streams? River Res Appl. 2017;33(10):1606–17

In any case, we have tried to avoid overstatements along the discussion section in the new version of the manuscript.

**2. Reviewer comment: Language in several parts of the discussion is too conclusory regarding causation for an observational study. Please restate to reflect the correlative nature of the study and analyses.**

Following the reviewer’s suggestions, we have reworded several sentences of the discussion section to make them less conclusory and acknowledge the limitations of our study.
