# Supplementary material for: Agricultural impacts on streams near Nitrate Vulnerable Zones: A case study in the Ebro basin, Northern Spain
Source: PLoS One. 2019 Nov 8;14(11):e0218582. doi: 10.1371/journal.pone.0218582 (PMC6839874; doi:10.1371/journal.pone.0218582)
Supplement: Attachment — Submitted filename: Response to reviewers_20_10_2019.docx [file pone.0218582.s003.docx]

Dear Editor,

Once more we would like to thank you and the reviewers for the new revisions and the valuable contributions to the manuscript. Most comments have been incorporated without discussion. Below you can find our answers to comments that required some explanation.

**EDITOR**

**1. Reviewer comment: Throughout the text replace NO3 by NO3−**

Response: We have replaced NO3 by NO_3_^−^ at manuscript, and accordingly, at Supporting Information (as the other ions) and at figure 4.

**Reviewer 1**

**1. Reviewer comment: What variables were removed because of the correlation with other variables?**

Response: Concentration of SO_4_^2-^, Ca_2_^+^ and Mg_2_^+^, Components 1, 2 and 7 of IHF index, QBR-total and Upstream siliceous rocks (%)

**2. Reviewer comment: “Group 1 was characterized by taxa with short reproductive cycles”, “Group 2 was characterized by taxa with longer reproductive cycles”. This is wrong, if the studied trait represents the number of reproductive cycles per year, as for line 203. The asymmetric beanplots show that Group 1 has higher proportion of the modality “less than 1” and “1” compared to Group 2; and Group 2 has higher proportion of the modality “more than 1” compared to Group1. I´d recommend to describe them as mono- or semivoltine taxa (group 1) and polyvoltine taxa (group 2), rather than taxa with long/short reproductive cycle.**

Response: We are sorry for this mistake. According to reviewer recommendations, we have described taxa in the test as mono-, semi- or polyvoltine taxa, and we have accordingly added these terms in the Supporting Information.

**3. Reviewer comment: Check figure numbering. The text refers to the asymmetric beanplots as Figure 3, but it was uploaded as Figure 4. The dbRDA plot was upleaded as Figure 3, instead of Figure 4.**

Response: We uploaded now asymmetric beanplots as figure 3 and dbRDA plot as figure 4, as it is correct.

**Reviewer 3**

**1. Reviewer comment: “grazers” – This FFG probably reflects how different taxa are assigned (i.e., there is a separate category for “scrapers”), but an increase in “grazers” is at odds with “reducing substrate availability for grazing mayflies” as per smothering algae effects (L96). I´m not certain much can be done about this apparent contradiction, although the authors should be citing Rabeni et al. (2005) here and in the Discussion – and those researchers found that scrapers (presumably including grazers) decreased with increasing sedimentation. Note also that a decrease in shredders as per the expectation at L114-119 is at odds with what Rabeni et al. 2005 found; although Burdon et al. 2018 found that grazers (including scrapers) and shredders decreased across a landscape disturbance gradient whilst collectors become proportionally more abundant.**

Response: We have cited now Rabeni *et al*. (2005) and Burdon *et al*. (2018) in the introduction and in the discussion. Both studies are in line with our findings of scrapers decrease under sediment increase.

Regarding shredders, we are not sure why Rabení and Burdon find opposite patterns, but we have suggested the following: “The abundance of shredders was also reduced under agricultural intensification. As suggested by Burdon et al. [27], this is most likely due to degraded riparian habitats (Fig 5), since shredders do not seem to be directly affected by sediment deposition [33]”.

**2.** **Reviewer comment: define “agricultural” land here – i.e., arable cropping, etc. “Pasture” is mentioned at L332 which is also an agricultural land use**

Response: we have detailed that agriculture land refers to cultivated land when it is firstly cited in the manuscript.

**3. Reviewer comment: Table 2: Check “Leptophlebiidae” – I guess the # doesn´t belong here.**

Response: # symbol is correctly included here, since this family was significantly associated with group 1 according to IndVal analysis, but *Habroleptoides sp.* and *Habrophlebia sp.* were not significantly associated to any group according to this analysis, probably related to their lower densities.
